# Using plant circadian programs to optimize agrochemical use

**DOI:** 10.1111/nph.70346

**Published:** 2025-06-29

**Authors:** Gustavo Akio Ogasawara, Carlos Takeshi Hotta, Antony N. Dodd

**Affiliations:** ^1^ John Innes Centre, Norwich Research Park Norwich NR4 7RU UK; ^2^ Departamento de Bioquímica Instituto de Química, Universidade de São Paulo São Paulo SP 05508‐000 Brazil

**Keywords:** agrochemicals, *Arabidopsis thaliana*, chronobiology, circadian rhythms, herbicide

## Abstract

Agrochemicals play an important role in maximizing agricultural yields. One class of agrochemicals is herbicides, which are used for weed control and to kill the top growth of certain crops before harvest. We examine the influence of plant circadian regulation on herbicide effectiveness to exemplify how knowledge of the circadian clock could be mobilized to optimize agrochemical use. First, we briefly introduce the circadian clock, highlighting its role in plant fitness and regulating physiological and metabolic pathways. Second, we discuss principles of chronotoxicity and the range of herbicides across which agrochemical interventions might be optimized with knowledge of circadian rhythms. Using existing data, we find that a substantial number of pathways targeted by herbicides are subject to circadian clock regulation, opening the possibility that clock control of herbicide effectiveness could be widespread. Finally, we suggest potential practical applications, explaining how this could enhance resource use efficiency, reduce inputs, and mitigate environmental impacts.

## Resumo

Os agroquímicos possuem um papel importante na maximização da produção agrícola. Herbicidas são uma classe de agroquímicos usados no controle de plantas daninhas e no manejo de algumas culturas durante a fase de pré‐colheita. Nós investigamos a influência da regulação circadiana das plantas sobre a eficácia dos herbicidas, para mostrar como o relógio circadiano pode ser usado para se otimizar o uso de agroquímicos. Primeiro, introduzimos o relógio circadiano, destacando seu papel na adaptação das plantas ao ambiente e na regulação de vias fisiológicas e metabólicas. Em seguida, discutimos os princípios que regem a cronotoxicidade, e a variedade de herbicidas nas quais intervenções agroquímicas poderiam ser otimizadas com base no conhecimento dos ritmos circadianos. Descobrimos que um número significativo de vias‐alvo de herbicidas é regulado pelo relógio circadiano, sugerindo que o controle circadiano da eficácia dos herbicidas possa ser algo generalizado. Por fim, sugerimos potenciais aplicações práticas, explicando como elas poderiam aumentar a eficiência no uso de recursos, reduzir insumos e mitigar seus impactos ambientais.

**Table:** 

	Contents	
	Summary	2557
I.	Introduction	2557
II.	Concept of chronotoxicity within plant sciences	2558
III.	Circadian clock modulation of herbicide effectiveness	2558
IV.	Approaches to the timed application of agrochemicals	2560
V.	Conclusion and perspectives	2562
	Acknowledgements	2562
	References	2562

## I. Introduction

Plant circadian clocks align physiology, metabolism, and development with 24 h environmental cycles. This influences many processes, including growth, reproduction, photosynthesis, stomatal opening, metabolism, and responses to the abiotic and biotic environment (Millar, 2016). As many of these are agriculturally important, applying knowledge of circadian clocks to agriculture – known as chronoculture or circadian agriculture – represents an innovative field (Steed *et al*., 2021).

In plants, the circadian clock involves a cellular oscillator formed from several interlocked transcription–translation feedback loops, which also includes post‐translational mechanisms (Millar, 2016). The oscillator is adjusted by predictable environmental cues such as light and temperature, through the process of entrainment, to align the oscillator with environmental time. In turn, the oscillator is coupled to diverse processes by interaction of its components with regulators of gene expression and metabolism. Under constant environmental conditions, the oscillator produces a self‐sustaining free‐running period of *c*. 24 h. This *c*. 24 h period is relatively stable within a range of physiologically relevant temperatures (‘temperature compensation’) (Millar, 2016). Although it is informative to study circadian rhythms under constant conditions, the clock is an adaptation to 24 h environmental fluctuations and, therefore, determines the ‘phase relationship’ between biological processes and daily environmental fluctuations (Pittendrigh, 1993). Through this, the plant circadian clock is thought to confer a selective advantage (Dodd *et al*., 2005).

## II. Concept of chronotoxicity within plant sciences

In medicine, there is considerable interest in controlling the daily timing of drug treatments to maximize their effectiveness and minimize unintended effects (Dallmann *et al*., 2016). This harnesses the relationship between the circadian control of the activity of drug targets (or off‐targets) and the time of day of drug delivery, with evidence that this can enhance patient outcomes (Lévi *et al*., 1997; Dallmann *et al*., 2016). More than 50% of the most commonly used drugs in the United States have circadian‐regulated targets (Zhang *et al*., 2014), suggesting widespread potential to optimize drug use through knowledge of the patient's circadian rhythm (Lévi *et al*., 2024).

A comparable situation may exist in plants. The plant circadian clock coordinates the accumulation of a substantial proportion of the transcriptome (e.g. 33%, Romanowski *et al*., 2020), translatome (e.g. 39%, Bonnot & Nagel, 2021), and aspects of the phosphoproteome (338 phosphopeptides, Krahmer *et al*., 2022). This breadth of clock control means that chemicals applied to plants that affect metabolism or signaling can encounter targets having 24 h fluctuations, leading to circadian modulation of the response of the plant to the chemical. For example, when exogenous auxin is applied to *Arabidopsis thaliana* (Arabidopsis) at different times of day, the magnitude of the resulting signaling response is clock‐regulated (Covington & Harmer, 2007). Similarly, a chemical genetics study of the Arabidopsis circadian clock found that the magnitude of repression of certain clock transcripts by a small molecule (PHA767491) varies according to its application time in the 24‐h cycle (Uehara *et al*., 2019). This indicates that the circadian clock can modulate responses of plants to exogenous chemicals, which could include agrochemicals.

## III. Circadian clock modulation of herbicide effectiveness

Herbicides are used to manage weeds that compete with crops and to kill the top growth of some crops before mechanical harvest. They kill plants by interfering with essential biochemical pathways that occur widely across plant life. This includes photosynthetic inhibitors that often block electron transport and chemicals that interfere with metabolism and signaling. For example, the widely used herbicide glyphosate inhibits the enzyme 5‐enolpyruvyl‐shikimate‐3‐phosphate synthase within the shikimate pathway, which is necessary for the synthesis of a variety of essential molecules, including aromatic amino acids. Commercial herbicides often contain several active ingredients to increase their effectiveness and adjuvants to increase their absorption. Some herbicides have activity across many species, whereas others are selective (e.g. kill grasses only) through certain combinations of adjuvants, safeners, and modes of action.

Herbicide use presents a good model to study the potential for developing the concept of chronotoxicity in plants, because many herbicides have well‐characterized modes of action. Understanding herbicide chronotoxicity could provide opportunities to enhance their effectiveness and reduce waste. For example, some herbicides inhibit aspects of the light reactions of photosynthesis, which are controlled by the circadian clock (Hennessey & Field, 1991; Dodd *et al*., 2005). Therefore, a treatment with a transient effect that targets photosynthetic processes might be more effective when applied at times of greater photosynthetic capacity. Some commercial herbicides provide recommendations about optimum application times. For example, Gesparim (atrazine‐based) recommends use between 10:00 and 16:00 h, when humidity levels maximize its effectiveness, whereas many products do not specify the optimum application time (e.g. Syngenta ZAPP QI620 (glyphosate formulation) and Copalliance 2,4‐d (a synthetic auxin)).

Several studies have sought to understand the relationship between the time of application and herbicide effectiveness. We analyzed the scientific literature to identify the most‐studied herbicide active ingredients (Supporting Information Dataset S1). Using this set of most‐studied active ingredients, we found that herbicide effectiveness varies according to the time of day for several herbicide modes of action (Table 1) (Stopps *et al*., 2013; Sharkhuu *et al*., 2014; Aliverdi *et al*., 2020; Kalina *et al*., 2022). In circadian medicine, drugs with 24‐h cycles of effectiveness typically have short half‐lives (Lee *et al*., 2021), whereas the half‐life of chemicals used as herbicides can be less well defined. A challenge for understanding herbicide half‐lives is that degradation can depend on environmental microorganisms (Sviridov *et al*., 2015; Bao *et al*., 2024).

**Table 1:** The most studied herbicides in the past 5 years, by scientific publication.

Herbicide	Mode of action	Target metabolic pathway	Chronotoxicity potential	References
Glyphosate	Inhibition of enolpyruvyl shikimate phosphate synthase	Shikimate pathway	High	Belbin *et al*. (2019); Kalina *et al*. (2022)
2,4‐d	Auxin mimics	Multi pathways	High	Montgomery *et al*. (2017)
Atrazine	PSII inhibitors–serine 264 Binders	Photosynthesis	Low	Sellers *et al*. (2003)
Paraquat	PS I electron diversion	Photosynthesis	High	Montgomery *et al*. (2017)
Glufosinate	Inhibition of glutamine synthetase	Glutamine synthesis	High	Montgomery *et al*. (2017)

Data derived from Web of Science (Supporting Information Dataset S1) and concern a variety of species. We considered the potential to use knowledge of the circadian clock to tune the use of each of these herbicides according to: (1) literature indicating whether there is a time of day effect upon the outcome of herbicide application; and (2) whether the target metabolic pathway has rhythmic components.

One study tested directly the influence of the circadian oscillator upon a herbicide response. A proof‐of‐concept study using *A. thaliana* under controlled conditions found that inhibition by glyphosate of hypocotyl elongation had a 24‐h cycle, with inhibition being greatest immediately after dawn, and absent at dusk (Belbin *et al*., 2019). Under free‐running conditions, there was a circadian oscillation in this response to glyphosate, which was abolished in arhythmic genotypes. These phenotypes partly extended to three other species (*Brassica napus*, *Sinapis arvensis*, and *Panicum miliaceum*), with some differences in the time of day of greatest glyphosate sensitivity compared with Arabidopsis (Belbin *et al*., 2019). Therefore, 24‐h cycles of glyphosate effectiveness reported under field conditions (Martinson *et al*., 2002) could arise from circadian regulation. Belbin *et al*. (2019) did not investigate potential roles for circadian regulation in glyphosate sensitivity under field conditions, where responses will be modulated by environmental fluctuations (e.g. the weather conditions) and changes in agrochemical spray interception through factors such as 24 h oscillations in leaf angle.

**Fig. 1:**
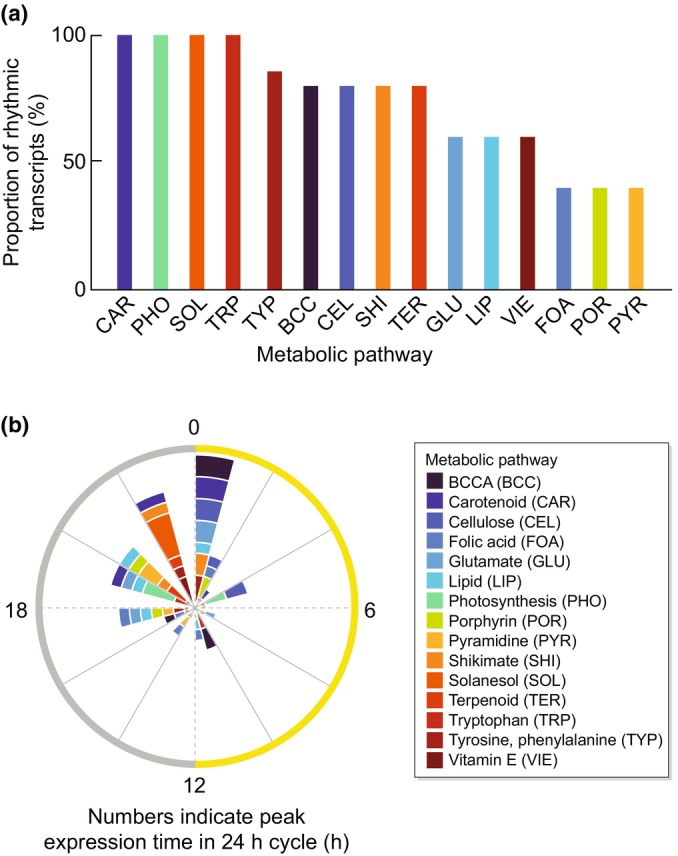
The metabolic targets of multiple herbicides are circadian‐regulated. (a) Five enzymes were selected within each of 15 metabolic pathways that are herbicide targets. The proportion of these enzymes that have a circadian rhythm in transcript abundance and ribosome binding (translation potential) was calculated. Only genes that had rhythmic transcript accumulation and rhythmic ribosome binding were categorized as rhythmic within this analysis. Colored bars represent the number of rhythmic transcripts for each pathway. Abbreviations are explained on the right‐hand side. (b) Under free‐running conditions, the time of maximum accumulation of rhythmic transcripts within each of the 15 target metabolic pathways. The radial axis represents the proportion (%) of transcripts. Yellow and gray shading on the circumferential axis indicates subjective day and night, respectively. Data derived from *Arabidopsis thaliana* were extracted using CAST‐R (Bonnot & Nagel, 2021) and Romanowski *et al*. (2020), with an enzyme called rhythmic, where *P* < 0.05 using MetaCycle to test for rhythmicity (Hughes *et al*., 2010) (Supporting Information Dataset S2). Numbers around the circular axis refer to the peak expression time (h), relative to entrained dawn, under free‐running conditions.

We were interested in the potential for circadian regulation of herbicide responses amongst the most common modes of action. We selected 15 metabolic pathways targeted by herbicides, based on all 25 described modes of action (Heap, 2025), choosing five enzymes from each. Where possible, we selected the molecular target of the herbicide and two enzymes upstream and two downstream within the pathway. Using published data from Arabidopsis (Romanowski *et al*., 2020; Bonnot & Nagel, 2021), we examined which of these enzymes underwent statistically significant circadian regulation of transcript abundance and translation (Dataset S2). We classified enzymes meeting both criteria as undergoing potential circadian clock control. This found that all the examined pathways contain enzymes with circadian regulation of gene expression (Fig. 1). Of these, 100% of enzymes examined within carotenoid synthesis, photosynthesis, solanesol biosynthesis, and tryptophan (TRP) synthesis pathways underwent circadian‐regulated expression, with > 50% circadian‐regulated in tyrosine, phenylalanine, branched‐chain amino acid, cellulose, shikimate, terpenoid, glutamate, lipid, and vitamin E metabolic pathways, and < 50% circadian‐regulated in folic acid, porphyrin, and pyramidine metabolic pathways (Fig. 1a).

**Fig. 2:**
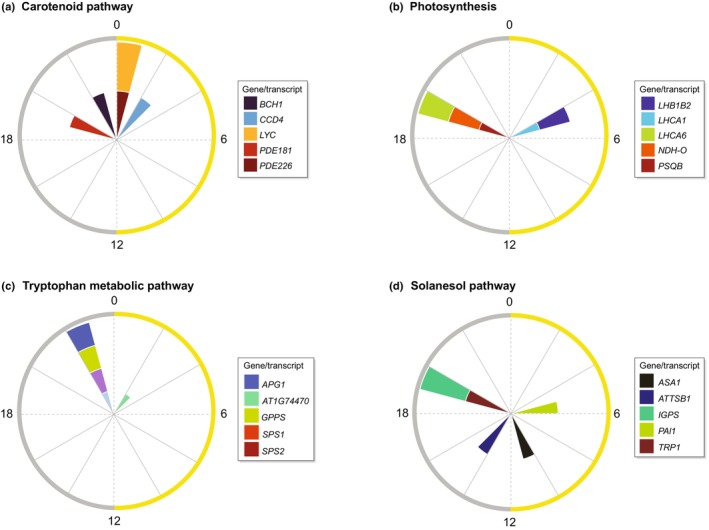
Metabolic targets of different herbicide modes of action have the greatest transcript accumulation at distinct times. Data for five selected genes from (a) carotenoid pathway, (b) photosynthetic pathways, (c) tryptophan metabolic pathway, and (d) solanesol pathway. Data derived from *Arabidopsis thaliana* were extracted using CAST‐R (Bonnot & Nagel, 2021) and Romanowski *et al*. (2020), with enzymes called rhythmic, where *P* < 0.05 using MetaCycle to test for rhythmicity (Hughes *et al*., 2010) (Supporting Information Dataset S2). Gene names correspond to abbreviated Arabidopsis Genome Initiative annotations. Numbers around the circular axes refer to the peak expression time (h), relative to entrained dawn, under free‐running conditions. Radial axes represent the proportion (%) of transcripts within the pathway. Yellow and black shading on circumferential axes indicates subjective day and night, respectively.

If the time of maximum or minimum expression of several pathway components corresponds with the time of greatest or least effectiveness of the pathway‐targeting herbicide, the phase of expression of these pathway components might influence the time(s) when the herbicide is most or least effective. Although many enzymes examined reached peak expression around subjective dawn or the latter part of subjective night (Fig. 1), there was some pathway‐specific variation in the peak expression times. For example, many components of the carotenoid synthesis pathway peak around subjective dawn (Fig. 2a), whereas photosynthesis, TRP, and solanesol synthesis components peak several hours before subjective dawn (Fig. 2b–d). The significance of this is that where components of each pathway have differing phases (Fig. 2), chemicals targeting each of these pathways could have different optimal application times.

If clock‐controlled expression of these enzymes causes rhythmic responses to different herbicide active ingredients, the potential for clock modulation of herbicide effectiveness could be extensive. However, this may overestimate or underestimate the importance of rhythms of transcript levels upon enzyme activity. Additionally, this analysis does not provide information about whether metabolic flux through each enzyme is clock‐controlled. This analysis is based on Arabidopsis, whereas in weed or crop species, there might be variation in the times of peak expression or activity. The data enabling this analysis were collected under free‐running conditions (constant light), with these regulatory patterns likely modified under 24‐h cycles of light and dark (including naturally fluctuating conditions). Finally, this analysis does not consider the pharmacokinetics of the herbicide because absorption, translocation, and detoxification likely have rhythmic components.

## IV. Approaches to the timed application of agrochemicals

Under field conditions, time‐specified agrochemical application presents challenges because spraying an entire crop with a chemical at the single time of greatest effectiveness is unfeasible. Certain activities, such as soil preparation and fertilization, can take weeks. Furthermore, field microenvironments can delay or advance the plant circadian clock (Dantas *et al*., 2021) and there are seasonal changes in clock dynamics under natural conditions (Nagano *et al*., 2019). These factors should not discourage the timed application of agrochemicals as a strategy, but do reflect gaps in our current knowledge, including solvable technological challenges. Thus, tuning agrochemical use with knowledge of the plant circadian clock is complex and nuanced.

**Fig. 3:**
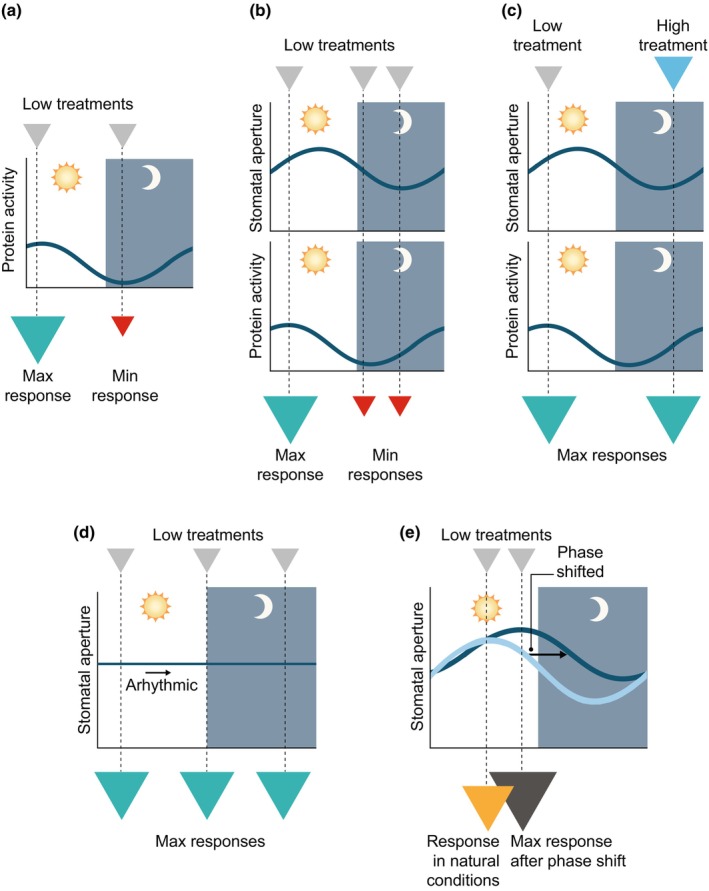
Conceptual approaches to optimize herbicide use through knowledge of plant circadian regulation. (a) The magnitude of response (colored arrows) to an identical herbicide treatment provided at two different times of day, where the response is determined by an oscillating factor involved in herbicide sensitivity, such as protein activity. (b, c) Potential outcome when two factors – stomatal aperture and target protein activity – act in combination to determine the magnitude of response to a herbicide. Here, (b) shows that identical herbicide application at different times produces varying magnitudes of response, whereas in (c) tuning the dose according to the sensitivity of the plant can produce a uniform response. (d, e) Manipulations to rhythmic processes in plants to adjust herbicide sensitivity. (d) Opening the stomata to override the effect of the clock upon herbicide entry eliminates time‐of‐day fluctuations in sensitivity. (e) Shifting the circadian phase with an entrainment cue (zeitgeber) to increase herbicide sensitivity. Small and large inverted arrows indicate relative magnitudes of herbicide input and efficacy. These are hypothetical examples, but suggest future research areas to use knowledge of circadian rhythms to optimize agrochemical use. Diagrams represent a 24‐h cycle, with white and gray sections indicating day and night, respectively.

Through some thought experiments, we suggest ways that herbicide chronotoxicity might be capitalized upon in agriculture. In a first example, we argue that factors with 24 h periodicity could shape the temporal response to an agrochemical (Fig. 3a). If the activity of a clock‐controlled target protein determines the effectiveness of the herbicide, the time of expression of this protein could direct the optimum time of herbicide application (Fig. 3a). Where a combination of two clock‐controlled factors influences herbicide effectiveness (e.g. a target protein and stomatal aperture), the situation becomes more complicated (Fig. 3b). Upon penetrating the leaf, if the active ingredient's target has greatest activity at an earlier time than the greatest stomatal aperture, the optimum treatment time might be shifted earlier (Fig. 3b). In an alternative scenario, knowledge of 24‐h fluctuations in sensitivity to the herbicide could be used to adjust the application rate according to time of day, to minimize agrochemical use (Fig. 3c). Here, a lower application rate is used at times of greater susceptibility (in this example, the morning), whereas a greater rate is used when the susceptibility is lower (here, the nighttime) (Fig. 3c). This is reminiscent of the circadian oscillation in the effective dose of glyphosate observed under controlled conditions (Belbin *et al*., 2019), and might be used to balance herbicide effectiveness against the need to minimize product use, to manage cost, and environmental protection.

In further examples, we propose that manipulation of the circadian oscillator or its outputs might allow the plant to be sensitized to an agrochemical. In the case of stomatal barriers to agrochemical uptake, an environmental or chemical manipulation that opens stomata might be used to override the control of stomatal aperture by the clock, allowing equally effective treatment regardless of the application time (Fig. 3d). Although this could be achieved through cell type‐specific clock gene overexpression (Hassidim *et al*., 2017; Simon *et al*., 2020), it could also use a manipulation such as a transient humidity modification or blue light treatment, delivered from advanced farm machinery. A more complex manipulation is to target the circadian entrainment pathways to temporarily shift the circadian phase, moving the rhythmic cellular targets of the agrochemical to a time of greater sensitivity (Fig. 3e). Where multiple factors confer herbicide sensitivity, these might be brought into the same phase to provide the greatest herbicide response for minimum dose. In a similar manner, it might be possible to engineer crops where the herbicide target assumes a different phase angle or altered entrainment compared with weeds. This could render the crop less sensitive to the herbicide because the most herbicide‐sensitive time for the weed would differ from the most sensitive time for the crop. A risk is that manipulations that perturb the phase could also have deleterious effects on agriculturally important traits, given that misalignment between the clock and environment impacts growth (Dodd *et al*., 2005).

Approaches such as these (Fig. 3) represent ideas that can be advanced by plant chronobiology research. Temporal responses to glyphosate are species‐dependent (Belbin *et al*., 2019) and aspects of circadian regulation in crops, including lettuce and wheat, can be cultivar‐specific (Higashi *et al*., 2014; Wittern *et al*., 2023), adding complexity. Furthermore, heterogeneity in the circadian phase caused by environmental heterogeneity (Dantas *et al*., 2021) means that continuous in‐field circadian phenotyping might help to tune herbicide dose to the circadian time. One potential approach is to use Chl fluorescence monitoring, which has been tested in glasshouses (Moriyuki & Fukuda, 2016), but it is more challenging to deploy outdoors. Perhaps glasshouse horticulture or vertical farms present good testbeds for such approaches, given the greater consistency of the growing environment.

## V. Conclusion and perspectives

Agrochemical use is controversial, and there is an urgent need for clean and safe agricultural practices (Nicolopoulou‐Stamati *et al*., 2016). However, agrochemicals represent part of the set of agricultural tools. The development of new herbicides should focus on using safe compounds with specific targets, which have minimal environmental impact. Although we used herbicides as an example, the concept of agrochemical chronotoxicity has the potential to extend beyond herbicides. For example, circadian programs occur in fungi, including plant pathogenic fungi such as *Botrytis cinerea* (Hevia *et al*., 2015). Furthermore, there is clock control of the activity, development, and migration of insects (Pittendrigh, 1967; Merlin *et al*., 2012), with evidence that insect clock control affects herbivory (Goodspeed *et al*., 2012). Therefore, the use of fungicides and insecticides might also be optimized through knowledge of the circadian clocks of their target species.

In the future, perhaps novel agrochemicals, such as sprays to deliver RNAi for pathogen control (Niu *et al*., 2021), might use knowledge of plant and pathogen circadian programs to produce optimal outcomes. Although we focused on herbicides as a specific example, understanding how circadian clocks regulate responses of crops to agrochemicals can also help align practices such as irrigation and fertilization according to biological time (Steed *et al*., 2021). We are not advocating for the use of agrochemicals, but argue that knowledge of how circadian rhythms shape their effectiveness can form part of optimizing their use to improve efficiency and environmental protection.

## Competing interests

None declared.

## Author contributions

GAO, CTH and AND conceived the study. GAO wrote the initial draft and conducted data acquisition and analysis. GAO, CTH and AND reviewed and edited the final manuscript.

## Disclaimer

The New Phytologist Foundation remains neutral with regard to jurisdictional claims in maps and in any institutional affiliations.

## Supporting information


**Dataset S1** Analysis of most‐represented herbicides within the scientific literature.

**Dataset S2** Analysis of circadian regulation of transcript accumulation across key herbicide modes of action, using published data from *Arabidopsis thaliana*.
Please note: Wiley is not responsible for the content or functionality of any Supporting Information supplied by the authors. Any queries (other than missing material) should be directed to the *New Phytologist* Central Office.

## Data Availability

The source data used within this article are published elsewhere. Data used in Figs 1 and 2 are within Dataset S2.
